# Clinical outcomes and complications in Latarjet versus free bone block procedures for anterior shoulder instability: a meta-analysis of comparative studies

**DOI:** 10.1007/s00590-025-04485-0

**Published:** 2025-08-31

**Authors:** Aleksander Sulkowski, Bavin Pathmaraj, Dillan Dhanak, Victor Yan-Zhe Lu, Peter Domos

**Affiliations:** 1 King’s College London, London, UK; 2 University College London, London, UK; 3 Chinese University of Hong Kong, Hong Kong, China; 4 Present Address: Royal Free London NHS Foundation Trust, London, UK; 5 Present Address: Chelsea and Westminster Hospital NHS Foundation Trust, London, UK; 6 West Hertfordshire Teaching Hospitals NHS Trust, Watford, UK

**Keywords:** Latarjet, Bone block, Shoulder instability, Patient-reported outcomes, Meta-analysis, Recurrence rate, Range of motion

## Abstract

**Purpose:**

The Latarjet procedure is thought to reduce recurrence by creating a dynamic restraint against instability, known as the “sling effect”. Conversely, free bone block (FBB) techniques provide stability through bony augmentation alone, without relying on the sling effect. This study compares the clinical outcomes of the Latarjet procedure and FBB techniques in adult patients to determine whether the sling effect offers a significant advantage in reducing recurrence rates.

**Methods:**

A systematic search of PubMed, Embase, and MEDLINE databases was conducted on 03/03/2025. Only comparative studies that included data on recurrence rate, patient-reported outcome measures (PROMs), range of motion, or reoperation rates were selected for analysis. We assessed the risk of bias using RoB 2 and ROBINS-I tools and performed a meta-analysis. PROSPERO ID: CRD42024517955.

**Results:**

Ten studies met the inclusion criteria (665 patients, mean age 29, mean follow-up 37 months) and underwent meta-analysis: three retrospective, four prospective cohort studies, and three randomised controlled trials. The meta-analysis demonstrated no significant differences between the Latarjet procedure and FBB techniques in recurrence rates of shoulder instability (RR 1.05; 95% CI 0.53–2.10; *P* = 0.87), PROMs (ASES, Rowe, SSV, WOSI, and VAS), or reoperation rate. No publication bias was detected (Egger’s *P* = 0.15). Regarding range of motion, no significant differences were observed in any of the parameters (abduction, external or internal rotation, and forward flexion). The limitations of our meta-analysis include high heterogeneity and serious risk of bias of studies.

**Conclusion:**

Our results suggest that FBB procedures are viable and safe alternative treatment options to the Latarjet technique.

**Level of evidence:**

III.

**Supplementary Information:**

The online version contains supplementary material available at 10.1007/s00590-025-04485-0.

## Introduction

Patient characteristics associated with glenohumeral bone loss and subsequent shoulder instability include male sex, young age, and competitive sports involvement [1]. Anterior shoulder instability is often linked with both glenoid rim deficiency and Hill-Sachs lesions. When such bony defects exist, it is common to see recurrent instability and deficits in shoulder function [2]. Usage of soft tissue stabilisation techniques for anterior shoulder instability has been associated with high recurrence rates [3]. Several theories exist as to why soft tissue stabilisation alone in the setting of critical bone loss leads to a high rate of recurrent instability. For instance, following bone loss, there is a reduction in glenoid contact area that in turn increases contact pressure. This is thought to produce increased shear forces on the repair, leading to high rates of failure [4]. The alternative treatment is reconstructing the glenoid rim by use of bone grafting procedures [5].

The Latarjet technique [6] is a popular bone block procedure that has been subject to variations but can broadly be divided into the classic and congruent arc (CA) methods. The classic method involves taking a coracoid graft and securing it so that its lateral border is flush with the glenoid articular surface [7]. The CA method involves rotating the coracoid graft 90° so that its inferior border is instead flush with the glenoid articular surface. It is theorised that the Latarjet procedure has an additional stabilising “sling effect” due to support from the repositioned conjoint tendon, which consists of the coracobrachialis muscle and the short head of the biceps [8]. As the coracoid is transferred and attached to the glenoid rim, the conjoint tendon forms a sling around the humeral head, particularly in the externally rotated and abducted positions [8]. The sling helps reduce the anterior translation of the humeral head by providing a compressive force, which is particularly helpful in throwing or overhead motions [8, 9].

In an effort to further improve stability in cases of glenoid bone loss, there are alternative techniques that focus on autologous or allogeneic bone grafts from alternative sites, thus preserving the native skeletal anatomy of the shoulder. These free bone blocks (FBB) include the distal clavicle autograft (DCA), the iliac crest bone graft (ICBG), and distal tibia allograft (DTA). The ICBG and other FBBs are often proposed in the setting of revision surgery where a primary Latarjet procedure has failed [10]. Surgeons might choose the Latarjet procedure due to concerns over complications at the graft donor site [11].

A recent meta-analysis has examined comparative studies of the Latarjet and ICBG procedures exclusively [12], while another meta-analysis which also incorporated the DTA approach has identified only four clinical studies [13]. Our study expands on this body of literature by incorporating a larger number of comparative studies, enabling an assessment of publication bias, and, for the first time, integrating data from a 5-year follow-up of the largest randomised controlled trial on this topic [14]. Therefore, this systematic review and meta-analysis aims to assess clinical outcomes, with a focus on recurrence rates, of the Latarjet procedure compared to alternative free bone block techniques for treating recurrent anterior shoulder instability with significant bone loss. We hypothesise that the dynamic sling effect of the Latarjet procedure results in a lower recurrence rate of instability.

## Methods

This review was conducted in accordance with the PRISMA guidelines [15] and has been registered in the PROSPERO database (ID: CRD42024517955). Although the original protocol planned to include both clinical and biomechanical studies, this review focuses exclusively on clinical outcomes, specifically the primary outcome of recurrence of anterior shoulder instability. Biomechanical studies were excluded post hoc to maintain focus on the main research objective.

### Search algorithm

A systematic search was initially performed on 23 February 2024 using three databases: embase (via Ovid), MEDLINE (via Ovid), and PubMed and repeated on 3 March 2025. The search strategy included variations of key terms such as ‘Latarjet’, ‘instability’, and ‘bone transplantation’ amongst others; a detailed search strategy is shown in Supplementary Table S1. All studies found in our search were imported into PICO Portal (https://www.picoportal.org/) and deduplicated. Two independent reviewers, blinded from each other’s decisions, completed title and abstract screening, followed by full-text screening, according to the inclusion and exclusion criteria. Agreement between reviewers was recorded at each stage: title and abstract screening (99%) and full-text screening (91%). Disagreements between reviewers were resolved by a third author. In cases of ambiguity, issues were discussed collectively by the review team until consensus was achieved. Criteria for inclusion included: adult patients aged 18 years or older, minimum clinical follow-up duration of 12 months, and studies directly comparing Latarjet and FBB procedures. Exclusion criteria and further details are provided in Supplementary Table S2. The criteria were based on the PICOS model (Population, intervention, comparison, outcome, and study design) [16]. The PRISMA diagram was generated using the PRISMA2020 Shiny app and modified [17].

### Data extraction

Data were extracted from included studies by two blinded independent reviewers and compiled into a standardised Excel spreadsheet. The following categories of data were extracted from each study:

1. *Study characteristics* design, level of evidence, follow-up duration, and patient count
2. *Patient demographics* age, gender, body mass index, hand dominance, mean glenoid bone loss, and whether it was a primary or revision procedure
3. Type of FBB procedure compared to Latarjet
4. Rate of recurrence
5. Range of motion (ROM)
6. Patient-reported outcome measures (PROMs)
7. Radiographic outcomes

### Data analysis

Meta-analysis was conducted using RStudio for quantitative results in clinical studies that were deemed comparable, such as PROMs, ROMs, and complication rates. To pool effect sizes of continuous variables, the standard mean difference (SMD) was used, which was reported with 95% confidence intervals and *P*-values. For discrete variables, the relative risk (RR) was reported. Due to foreseen heterogeneity, a random-effects model was used. To assess heterogeneity, the *I*^2^ statistic and Cochran’s *Q* test were used. When the standard deviation (SD) was not reported, the estimator provided by Wan et al. was utilised [18]. When data were missing, the corresponding author of the study was contacted to retrieve them. Statistical significance was set at *P* < 0.05.

### Risk of bias

For all included papers, a risk of bias assessment was conducted by two independent reviewers and subsequently verified by another author. For non-randomised studies, we employed the Cochrane ROBINS-I tool [19], while randomised controlled trials were assessed using the RoB 2 tool [20]. The risk of bias plots was created using *robvis* [21].

## Results

### Study selection

**Fig. 1:**
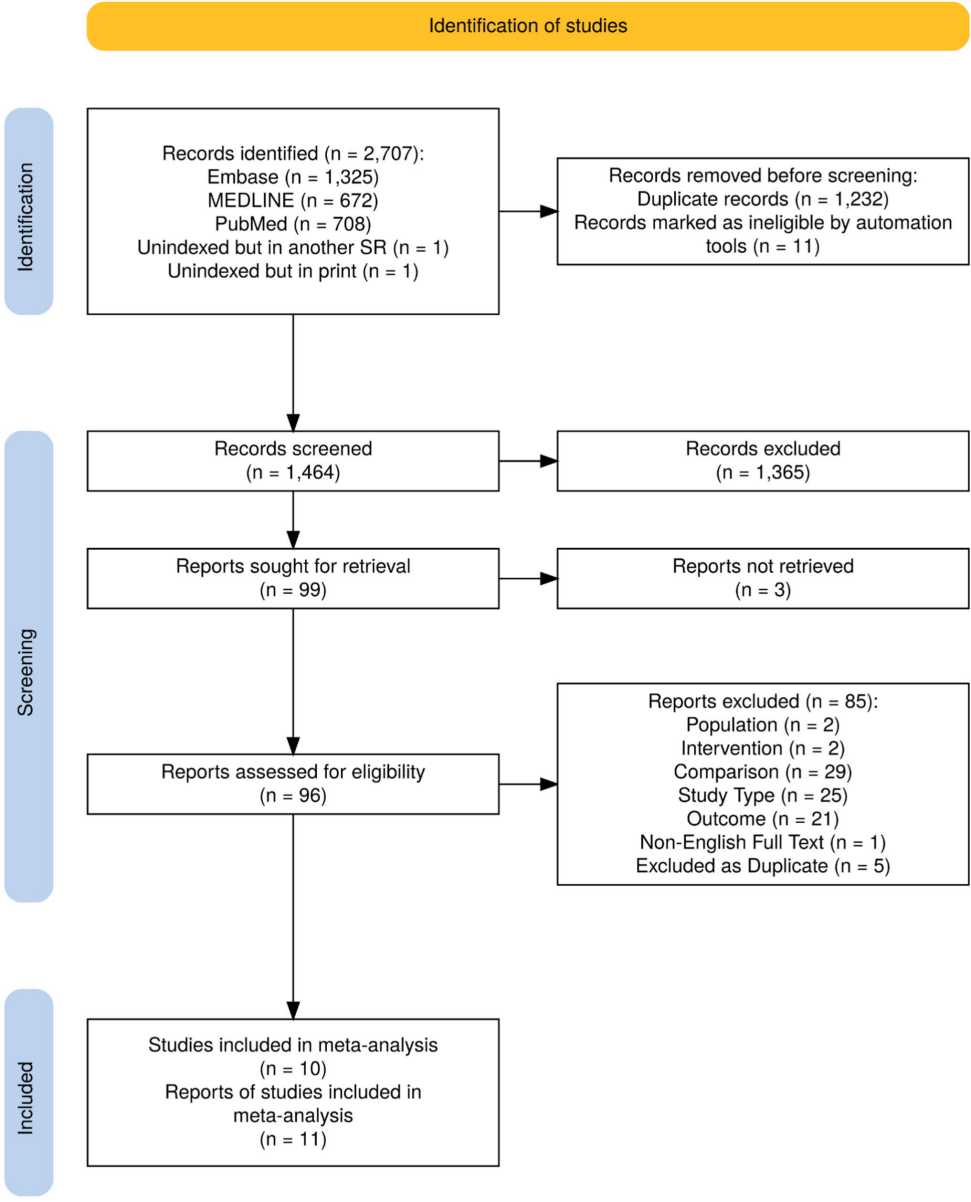
PRISMA flow diagram illustrating the study selection process. *PRISMA* preferred reporting items for systematic reviews and meta-analyses

We identified a total of 2705 studies through comprehensive database searches and added two relevant articles which have not been indexed in the databases. We removed 1243 duplicate/ineligible records prior to abstract screening and reviewed 99 full-text articles. Of those, 11 met inclusion criteria for the meta-analysis [14, 22–31], with Schulz et al. [14] reporting updated study follow-up data for a previous article by Moroder et al.[22] We utilised the updated data from Schulz et al. [14] where possible. This is summarised in Fig. 1.

### Studies characteristics

We evaluated three retrospective [26–28] and four prospective [23–25, 30] cohort studies, and three randomised controlled trials [14, 22, 29, 31] (Supplementary Table S3). A total of 665 patients were operated with one of the variants of the Latarjet procedure or with an alternative FBB. Two hundred and eighty-six Latarjet patients and 287 FBB patients were followed-up at a mean time of 37 months (range: 12–111 months). For the Latarjet procedure, eight studies used an open technique [14, 22–24, 27–31], while three studies employed an arthroscopic technique [25, 26, 30], where Delgado et al. had both open and arthroscopic Latarjet subgroups [30]. In contrast, for the FBB procedures, five studies utilised open techniques [14, 22–24, 27, 29], and five studies opted for arthroscopic techniques [24–26, 28, 30]. Studies involving the J-bone graft used the technique described by Auffarth et al. [32], while the technique by Taverna et al. [33] was commonly employed for arthroscopic ICBG. The average age was 27 years in the Latarjet cohort and 28 years in the FBB cohort. In the Latarjet group, 86% of patients were male, while in the FBB group, 91% were male. 69% of participants were operated on the dominant limb [24, 27–30]. Another three studies reported the number of previous shoulder surgeries per patient, with a mean of 0.76 [22, 23, 25]. The mean number of preoperative instability episodes was 14 in the Latarjet group and 19 in the FBB group [22, 25, 28]. 39% of Latarjet and 43% of FBB operations were revision procedures. Most studies reported glenoid bone loss, which averaged at 22%.

### Recurrence of instability

**Fig. 2:**
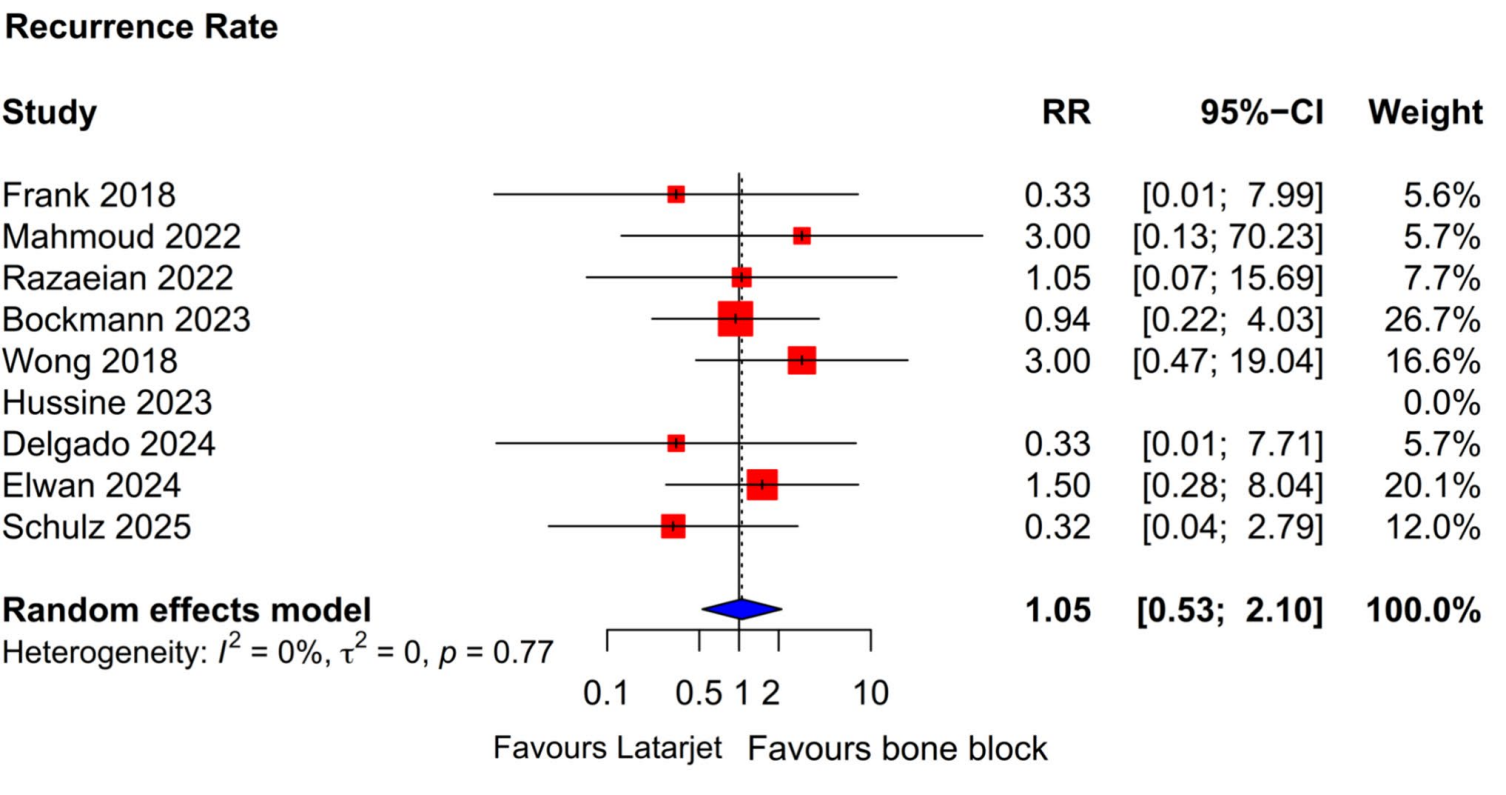
Forest plot showing pooled effect estimates for recurrence of shoulder instability. *CI* confidence interval, *RR* relative risk

Based on pooled estimates from eight studies, the instability recurrence rate was comparable between Latarjet and FBB groups (RR 1.05; 95% CI 0.53–2.10; *P* = 0.87) (Supplementary Table S4) (Fig. 2). Hussine et al. [31] reported no recurrence in either group while Carbone et al. [27] excluded any patients with instability postoperatively. Seven studies [14, 24–26, 28–30] specified the type of instability and the number of events: dislocation (Latarjet: 7; FBB: 6), subluxations (Latarjet: 3; FBB: 4), and apprehensions (Latarjet: 4; FBB: 5).

### Clinical outcome scores

**Fig. 3:**
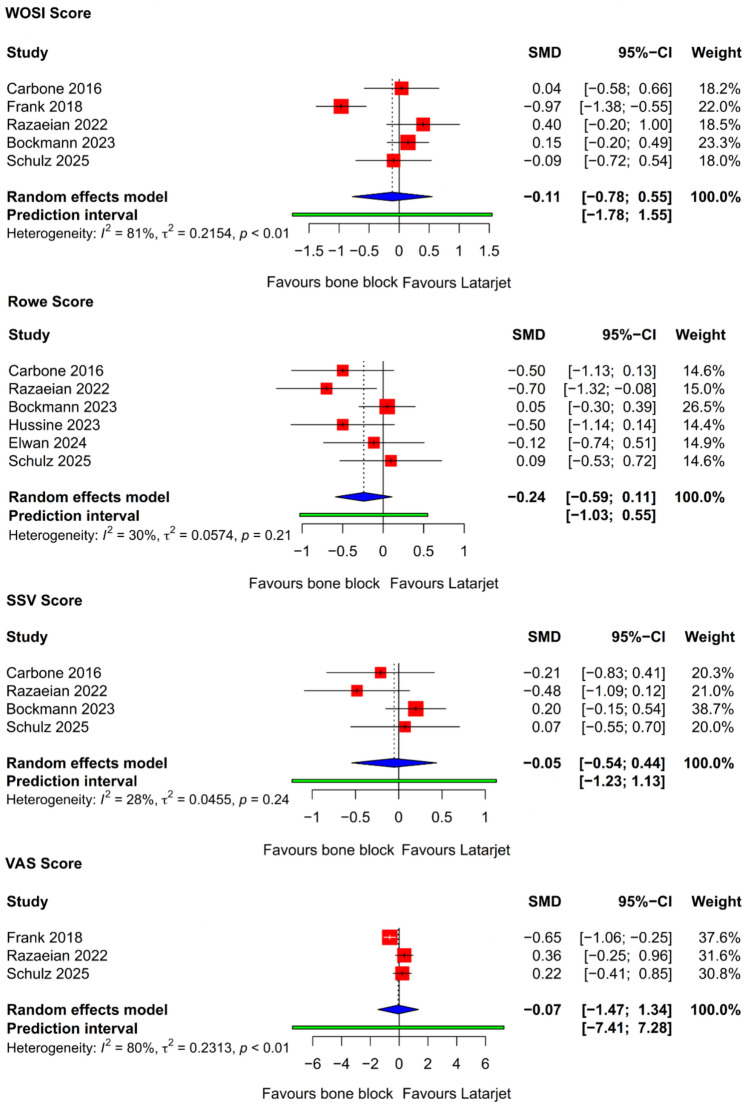
Forest plots displaying pooled effect sizes for patient-reported outcome measures: WOSI, Rowe score, SSV, VAS. *CI* confidence interval, *SMD* standard mean difference, *SSV* subjective shoulder value; *WOSI* Western Ontario shoulder instability index; *VAS* visual analogue scale

We evaluated PROMs by examining four key measures: the Western Ontario shoulder instability index (WOSI), the Rowe score, the subjective shoulder value (SSV), and the visual analogue scale (VAS) for pain (Supplementary Table S5) (Fig. 3). Overall, this analysis reveals the lack of significant differences in WOSI, Rowe, SSV, and VAS scores between patients undergoing the Latarjet procedure and FBB, indicating comparable outcomes.

### Range of motion

**Fig. 4:**
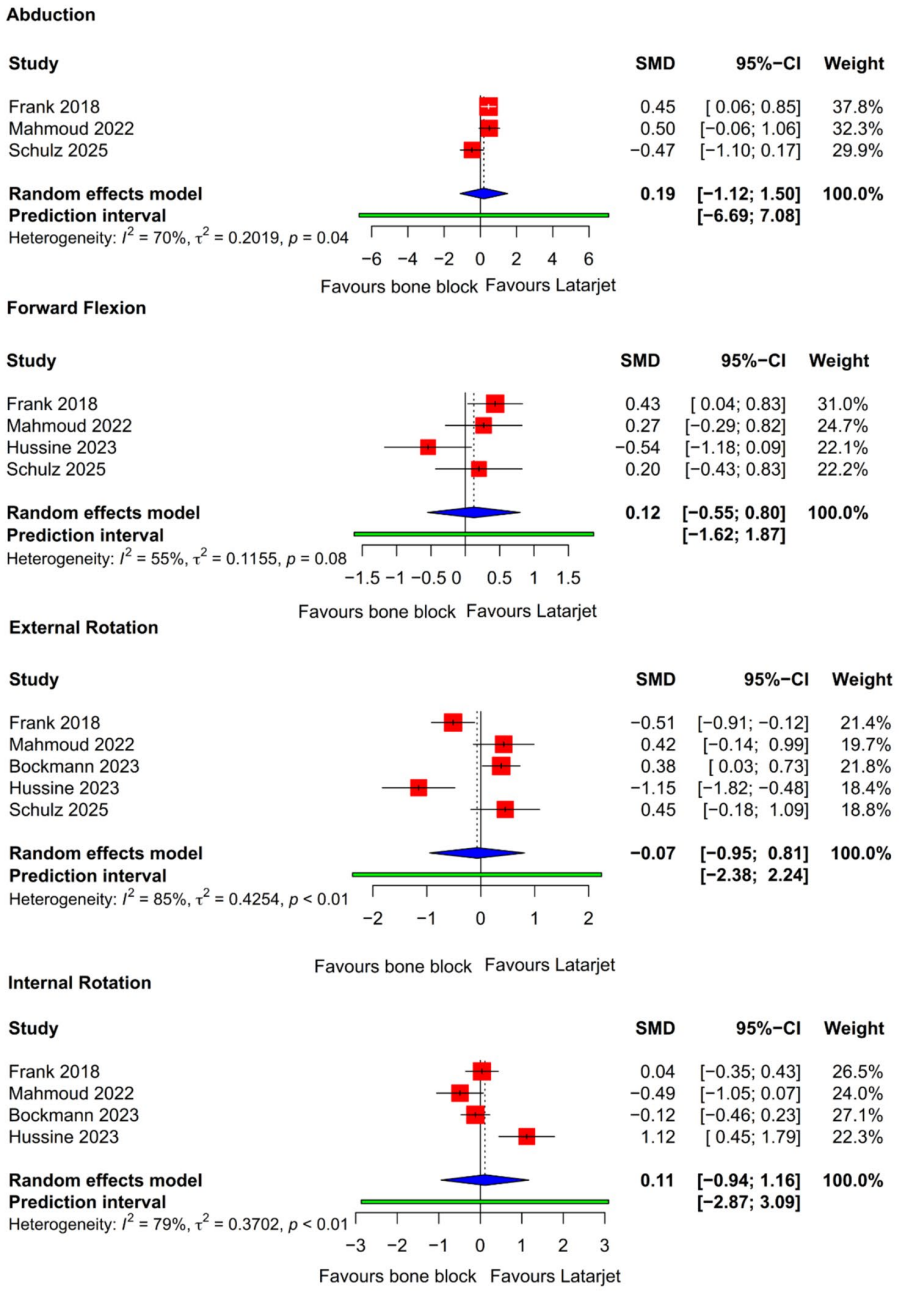
Forest plots showing pooled differences in shoulder range of motion: abduction, forward flexion, external rotation, and internal rotation. *CI* confidence interval; *SMD* standard mean difference

A total of five studies have reported a range of motion parameter [14, 23–25, 31]. There were no statistically significant differences in abduction, forward flexion, external rotation, or internal rotation (Supplementary Table S6) (Fig. 4).

### Adverse outcomes

**Fig. 5:**
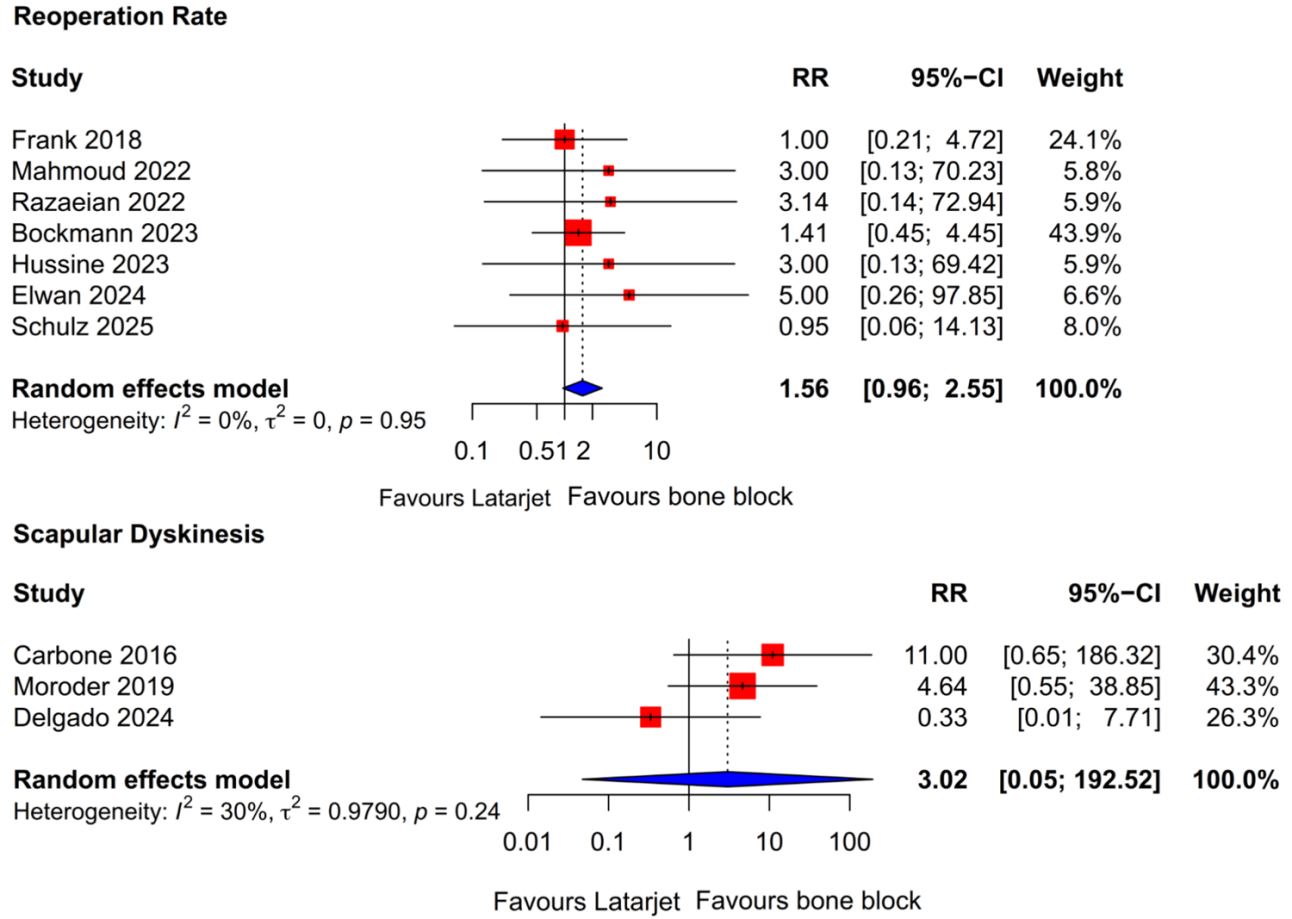
Forest plots showing pooled risk estimates for adverse outcomes: reoperation rate and scapular dyskinesis rate. *CI* confidence interval; *RR* relative risk

In the assessment of postoperative adverse outcomes, our meta-analysis encompassed reoperation rates and incidence of scapular dyskinesis. The difference in reoperation rate was not statistically significant (RR 1.56; 95% CI 0.96–2.55; *P* = 0.07) as was the difference in scapular dyskinesis rate (RR 3.02; 95% CI 0.05–192; *P* = 0.37) (Supplementary Table S7) (Fig. 5).

Mahmoud et al. [24] and Delgado et al. [30] were the only studies to report the graft resorption rates. The former observed resorption in 2 patients (8%) in the FBB group, with no cases in the Latarjet group. The latter reported more than 20% resorption in 75% of ICBG patients compared to only 10% in Latarjet patients. Only Schulz et al. provided data on the incidence of arthritis or the progression of osteoarthritis from pre to postoperative stages [14].

Razaeian et al. [28] and Hussine et al. [31] provided conflicting data on operating time. The former observed an average of 100 min (SD: 30.2) for the Latarjet procedure, compared to 118 min (SD: 33.5; *P* = 0.04) for the arthroscopic ICBG technique. In contrast, the former reported 77 min (SD: 10.19) for Latarjet and 73 min (SD: 10.56; *P* = 0.18) for ICBG. Razaeian et al. [28] also revealed that the time to return to sports was nearly identical between the groups, with the Latarjet group averaging 28 weeks (SD: 18.4) and the arthroscopic ICBG group 27 weeks (*P* = 0.8), suggesting no significant difference in recovery duration. The study by Mahmoud et al. [24] addressed the return to sports rate in overhead sports (e.g., handball, basketball, and volleyball), where 24 out of 25 patients in the Latarjet group and all 25 in the ICBG group successfully resumed their activities at the same level. This is corroborated by Delgado et al. [30] who reported the *P*-value of 0.18 for the return to sport rate between Latarjet and ICBG techniques.

In terms of patient satisfaction, measured via a 4-point Likert scale, Razaeian et al. [28] observed high satisfaction rates for both procedures; 86% of Latarjet patients and 82% of arthroscopic ICBG patients reported being 'very satisfied.' Moroder et al. [22] evaluation, using a 0–5 scale, also reflected similar satisfaction levels, with the Latarjet and ICBG procedures scoring an average of 4.9 (SD: 0.3) and 4.8 (SD: 0.8; *P* = 0.8), respectively. High satisfaction in both techniques was maintained at the 5-year follow-up [14].

### Risk of bias

**Fig. 6:**
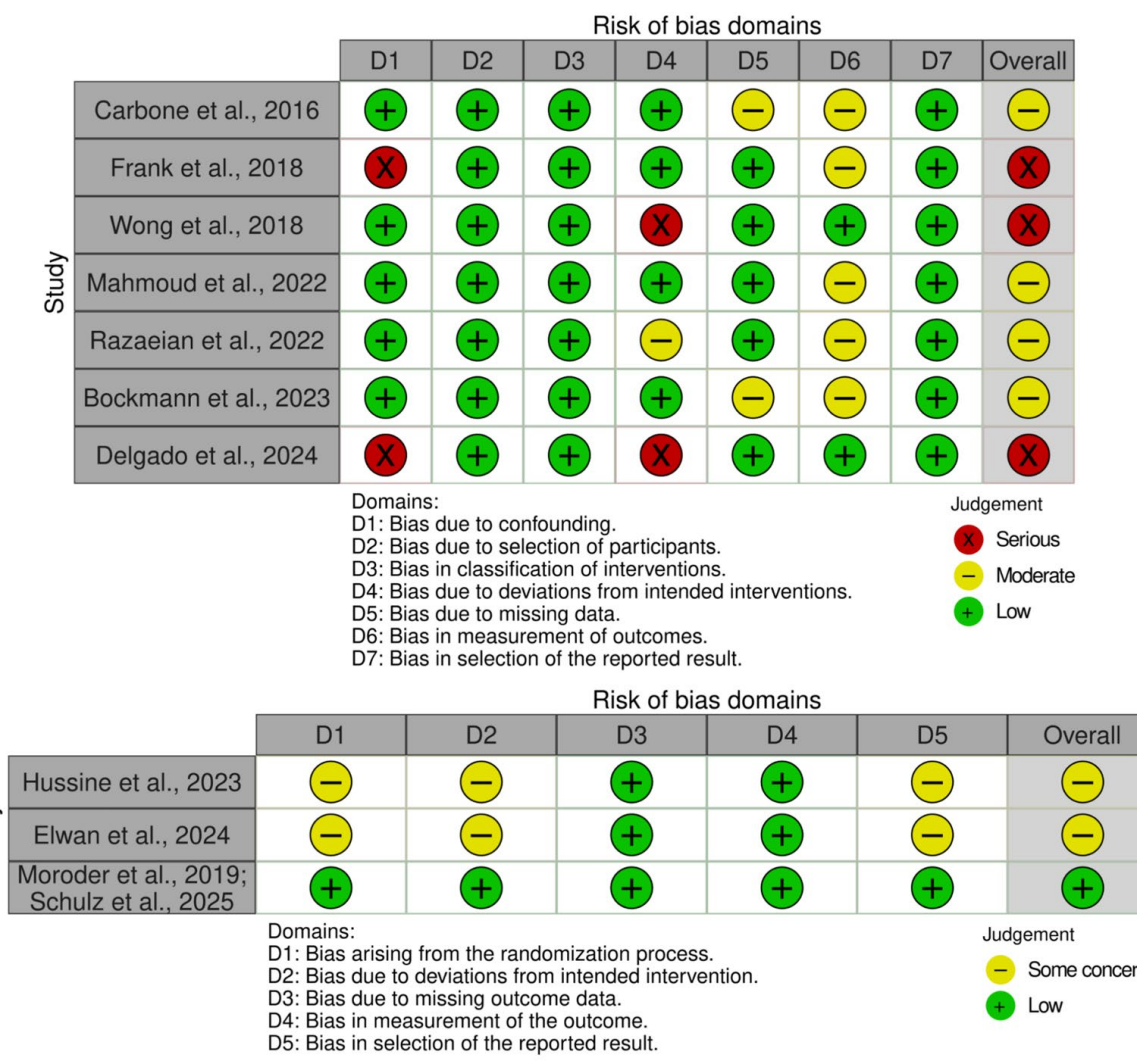
Risk of bias assessment conducted using the ROBINS-I tool for non-randomised studies and the RoB 2 tool for randomised controlled trials

Three studies [23, 26, 30] had serious risk, six studies [24, 25, 27–29, 31] had moderate risk, and only one study [14, 22] had low risk of bias (Fig. 6). Overall follow-up times were reported in all studies, with four studies[24–26, 28] presenting follow-up times for both the Latarjet and comparison bone block groups individually. In Razaeian et al. [28], the Latarjet group had a significantly longer follow-up time. Five studies[24, 27–30] reported the percentage of operations that took place on the patient's dominant hand. The number of preoperative instability episodes was reported in five studies [22, 25, 28, 29, 31].

**Fig. 7:**
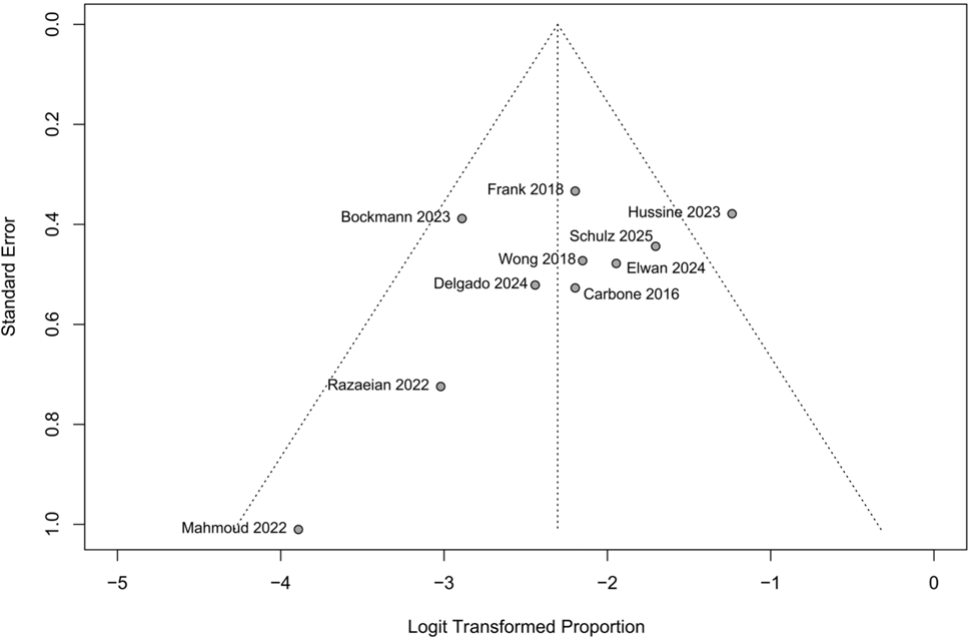
Funnel plot assessing potential publication bias

It is important to recognise that some studies introduced deviations from intended interventions that may have biased outcomes against the Latarjet procedure. For example, in Delgado et al. [30], remplissage was frequently added in the FBB group but not in the Latarjet group, and additional capsulolabral procedures varied across open and arthroscopic approaches. Similarly, in Wong et al. [26], a Bankart repair was performed in the DTA group but not in the Latarjet group. In Razaeian et al. [28], the AICBG technique was changed during the trial from as described by Scheibel et al. [34] to as described by Taverna et al. [33] due to theoretical concerns about potential risks to surrounding anatomical structures. We found no evidence of publication bias due to a lack of asymmetry on the funnel plot and an Egger’s test of the intercept *P*-value of 0.15 (Fig. 7).

## Discussion

### Key findings

This systematic review and meta-analysis of comparative studies found no significant differences between the Latarjet procedure and FBB techniques in terms of recurrence rate, PROMs, range of motion, reoperation rate, and the rate of scapular dyskinesis. In the studies, we retrieved the FBB procedures included ICBG and DTA transfers. These findings suggest that both procedures may offer comparable clinical efficacy in the treatment of recurrent anterior shoulder instability over the short to mid-term.

The lack of a statistical difference in the recurrence rate aligns with a previous meta-analysis by Gilat et al. [35], which reported primarily on non-comparative studies, and two recent comparative-only meta-analyses [12, 13]. Li et al. [12] reported statistically significant differences in external rotation and Rowe scores, however, these did not surpass the MCID, and our analysis, with a larger pool of studies, did not reproduce those findings. Our results in terms of ranges of motion and PROMs are in agreement with Gilat et al. [35] and Hao et al. [13] By focusing exclusively on comparative studies and incorporating longer follow-up data, such as the 5-year results from Schulz et al. [14], our review contributes additional clarity to the evolving evidence base comparing these two techniques.

Beyond headline outcomes, procedural nuances remain relevant. While the Latarjet procedure has long-standing support and predictable outcomes, it carries risks related to neurovascular injury and technical complexity, particularly if performed arthroscopically [36]. FBB techniques, particularly DTA, offer the theoretical advantage of anatomic glenoid restoration; however, donor site morbidity remains a concern with ICBG, while oversized DTA grafts may be associated with resorption due to Wolff’s law [26]. Despite these differences, our findings did not reveal a measurable impact on reoperation rates or scapular kinematics, suggesting that such concerns may be mitigated in well-selected cases. Clinically, this equivalence implies that surgeons can tailor their choice to patient-specific factors, like bone loss severity or tolerance for additional surgical sites, without sacrificing mid-term outcomes, provided they have adequate expertise.

The lack of difference in recurrence rates, defined in our analysis as a composite of dislocations and subluxations, invites further investigation into the stabilising mechanisms of these procedures. Both Latarjet and FBB techniques effectively prevent instability as we measured it; yet, their ability to address distinct instability subtypes remains uncertain due to inconsistent reporting. By grouping dislocations and subluxations, our analysis masked potential differences in these failure modes and excluded apprehension, a subtler instability marker. This fuels our call for future studies to stratify recurrence into dislocation, subluxation, and apprehension. Such granularity could uncover biomechanical advantages, enabling more precise patient selection and technique refinement for specific instability profiles.

Notably, only one included study [14] reported the secondary osteoarthritis rate despite its known association with anterior shoulder instability [37]. This gap limits our ability to assess long-term joint preservation, which is a critical consideration given the young, active population typically affected. Similarly, detailed and specific patient-centred outcomes like postoperative pain, recovery time, or satisfaction were underreported, leaving unanswered questions about the lived experience of each procedure. Addressing these in future research would provide a more holistic comparison, particularly as osteoarthritis could emerge as a differentiator over decades rather than years.

### Limitations

Our findings should be interpreted cautiously due to several limitations. Most included studies were non-randomised, which increases the risk of confounding bias due to potential systematic differences between groups. Preoperative characteristics varied across studies, with some reporting more instability episodes in the FBB group, indicating inconsistent case matching. Significant differences in glenoid bone loss were noted in certain studies; for example, Delgado et al. [30] found greater bone loss in the open Latarjet group, while Frank et al. [23] reserved DTA for patients with > 25% bone loss or failed prior Latarjet procedures, inherently selecting for more severe cases. Moreover, several outcomes, such as scapular dyskinesis, were assessed in only a few studies with limited sample sizes. This suboptimal information size reduces the certainty and generalizability of these specific findings.

Additional factors such as surgeon experience, technical variations, differing rehabilitation protocols, and variability in outcome assessment timing likely contributed to the substantial heterogeneity observed. Recurrence rates, in particular, are time-dependent and tend to increase with longer follow-up, so pooling studies with varying follow-up durations may lead to either overestimation or underestimation of the true recurrence risk. Unfortunately, these important variables were not consistently reported, limiting our ability to adjust for their effects. For example, in Wong et al. [26], a Bankart repair was performed in the DTA group but not in the Latarjet group; this difference reflects typical surgical strategy, as the Latarjet procedure achieves stability through bony augmentation and a dynamic sling effect, often eliminating the need for a separate Bankart repair. Nonetheless, these inconsistencies, particularly when additional stabilising procedures were applied selectively to non-Latarjet groups, may introduce bias against the Latarjet procedure.

On a positive note, formal assessment showed no significant publication bias, facilitated by the growing number of comparative studies. However, since the number of studies approached the minimum threshold for reliable bias detection, this finding should also be interpreted with some caution.

## Conclusions

This meta-analysis of comparative studies indicates that both the Latarjet and FBB procedures may provide similar outcomes in terms of recurrence, function, and range of motion in the management of anterior shoulder instability. However, the overall certainty of this conclusion is limited by the high risk of bias across included studies, particularly due to methodological heterogeneity and variable preoperative characteristics, which might be skewed against Latarjet and its dynamic sling effect. Although each technique presents unique technical considerations and biological implications, these did not translate into statistically significant differences in clinical endpoints within the follow-up periods assessed. To improve the quality of evidence, future research should prioritise long-term, multicentre randomised controlled trials with standardised outcome measures, stratified reporting of recurrence types at different time points, and inclusion of radiographic endpoints such as secondary osteoarthritis. Until then, both surgical approaches remain valid, with the choice best guided by patient-specific factors and surgical expertise.

## Supplementary Information

Below is the link to the electronic supplementary material.

Supplementary file1 (DOCX 16 kb)

Supplementary file2 (DOCX 18 kb)

Supplementary file3 (DOCX 31 kb)

Supplementary file4 (DOCX 26 kb)

Supplementary file5 (DOCX 23 kb)

Supplementary file6 (DOCX 18 kb)

Supplementary file7 (DOCX 26 kb)

## Data Availability

No datasets were generated or analysed during the current study.
