# Supplementary material for: Clinical outcomes and complications in Latarjet versus free bone block procedures for anterior shoulder instability: a meta-analysis of comparative studies
Source: Eur J Orthop Surg Traumatol. 2025 Aug 31;35(1):371. doi: 10.1007/s00590-025-04485-0 (PMC12399734; doi:10.1007/s00590-025-04485-0)
Supplement: Supplementary file 1 — Supplementary file1 (DOCX 16 kb) [file 590_2025_4485_MOESM1_ESM.docx]

| **Embase (via Ovid)** |
| --- |
| 1 exp Humans/  2 ("*Latarjet*" or "*Bristow*" or "sling*" or (coracoid adj5 trans*)).mp. [mp=title, abstract, heading word, drug trade name, original title, device manufacturer, drug manufacturer, device trade name, keyword heading word, floating subheading word, candidate term word]  3 exp Joint instability/  4 exp Shoulder dislocation/  5 exp Shoulder joint/pp  6 ("instabilit*" or "dislocat*" or "subluxat*" or "apprehens*").mp. [mp=title, abstract, heading word, drug trade name, original title, device manufacturer, drug manufacturer, device trade name, keyword heading word, floating subheading word, candidate term word]  7 ((loss* adj4 glenoid) or (defect* adj4 glenoid) or (defect* adj4 (bone* or bony)) or (loss* adj5 bone*)).mp. [mp=title, abstract, heading word, drug trade name, original title, device manufacturer, drug manufacturer, device trade name, keyword heading word, floating subheading word, candidate term word]  8 3 or 4 or 5 or 6 or 7  9 ("ICBGT" or "iliac crest*" or "coracoid*" or "J-bone*" or "J bone*" or "distal tibia*" or "distal clavicle*").mp. [mp=title, abstract, heading word, drug trade name, original title, device manufacturer, drug manufacturer, device trade name, keyword heading word, floating subheading word, candidate term word]  10 exp Bone transplantation/  11 exp Transplantation, autologous/  12 exp Transplantation, homologous/  13 exp Autografts/  14 exp Allografts/  15 exp Grafting, Bone/  16 "*graft*".mp.  17 "bone block*".mp.  18 ("augment*" or "reconstruct*" or "transplant*").mp. [mp=title, abstract, heading word, drug trade name, original title, device manufacturer, drug manufacturer, device trade name, keyword heading word, floating subheading word, candidate term word]  19 9 or 10 or 11 or 12 or 13 or 14 or 15 or 16 or 17 or 18  20 1 and 2 and 8 and 19 |

**Supplementary Table S1** Detailed search strategies. We searched Embase, MEDLINE and PubMed databases. No language restrictions were applied. Publication period was unrestricted. Unpublished studies were not sought.

| **MEDLINE (via Ovid)** |
| --- |
| 1 exp Humans/  2 ("*Latarjet*" or "*Bristow*" or "sling*" or (coracoid adj5 trans*)).mp. [mp=title, book title, abstract, original title, name of substance word, subject heading word, floating sub-heading word, keyword heading word, organism supplementary concept word, protocol supplementary concept word, rare disease supplementary concept word, unique identifier, synonyms, population supplementary concept word, anatomy supplementary concept word]  3 exp Joint instability/  4 exp Shoulder dislocation/  5 exp Shoulder joint/pp  6 ("instabilit*" or "dislocat*" or "subluxat*" or "apprehens*").mp. [mp=title, book title, abstract, original title, name of substance word, subject heading word, floating sub-heading word, keyword heading word, organism supplementary concept word, protocol supplementary concept word, rare disease supplementary concept word, unique identifier, synonyms, population supplementary concept word, anatomy supplementary concept word]  7 ((loss* adj4 glenoid) or (defect* adj4 glenoid) or (defect* adj4 (bone* or bony)) or (loss* adj5 bone*)).mp. [mp=title, book title, abstract, original title, name of substance word, subject heading word, floating sub-heading word, keyword heading word, organism supplementary concept word, protocol supplementary concept word, rare disease supplementary concept word, unique identifier, synonyms, population supplementary concept word, anatomy supplementary concept word]  8 3 or 4 or 5 or 6 or 7  9 ("ICBGT" or "iliac crest*" or "coracoid*" or "J-bone*" or "J bone*" or "distal tibia*" or "distal clavicle*").mp. [mp=title, book title, abstract, original title, name of substance word, subject heading word, floating sub-heading word, keyword heading word, organism supplementary concept word, protocol supplementary concept word, rare disease supplementary concept word, unique identifier, synonyms, population supplementary concept word, anatomy supplementary concept word]  10 exp Bone transplantation/  11 exp Transplantation, autologous/  12 exp Transplantation, homologous/  13 exp Autografts/  14 exp Allografts/  15 exp Grafting, Bone/  16 "*graft*".mp.  17 "bone block*".mp.  18 ("augment*" or "reconstruct*" or "transplant*").mp. [mp=title, book title, abstract, original title, name of substance word, subject heading word, floating sub-heading word, keyword heading word, organism supplementary concept word, protocol supplementary concept word, rare disease supplementary concept word, unique identifier, synonyms, population supplementary concept word, anatomy supplementary concept word]  19 9 or 10 or 11 or 12 or 13 or 14 or 15 or 16 or 17 or 18  20 1 and 2 and 8 and 19 |

| **PubMed** |
| --- |
| Humans[Mesh] AND ("*Latarjet*"[tw] OR "*Bristow*"[tw] OR "sling*"[tw] OR "coracoid trans*"[tw] OR "coracoid process trans*"[tw]) AND ("Joint instability"[Mesh] OR "Shoulder dislocation"[Mesh] OR "shoulder joint/physiopathology"[Mesh] OR "instabilit*"[tw] OR "dislocat*"[tw] OR "subluxat*"[tw] OR "apprehens*"[tw] OR "glenoid loss*"[tw] or "glenoid defect*"[tw] OR "bone* defect*"[tw] OR "bony defect*"[tw] OR "bone* loss*"[tw]) AND ("ICBGT"[tw] OR "iliac crest*"[tw] OR "coracoid*"[tw] OR "J-bone*"[tw] OR "J bone*"[tw] OR "distal tibia*"[tw] OR "distal clavicle*"[tw] OR "Bone transplantation"[Mesh] OR "Transplantation, autologous"[Mesh] OR "Transplantation, homologous"[Mesh] OR "Autografts"[Mesh] OR "Allografts"[Mesh] OR "Grafting, Bone"[Mesh] OR "bone block*"[tw] OR "*graft*"[tw] OR "augment*"[tw] OR "reconstruct*"[tw] OR "transplant*"[tw]) |
