# Supplementary material for: Clinical outcomes and complications in Latarjet versus free bone block procedures for anterior shoulder instability: a meta-analysis of comparative studies
Source: Eur J Orthop Surg Traumatol. 2025 Aug 31;35(1):371. doi: 10.1007/s00590-025-04485-0 (PMC12399734; doi:10.1007/s00590-025-04485-0)
Supplement: Supplementary file 2 — Supplementary file2 (DOCX 18 kb) [file 590_2025_4485_MOESM2_ESM.docx]

**Supplementary Table S2** Detailed inclusion and exclusion criteria used during study selection

|  | Inclusion | Exclusion |
| --- | --- | --- |
| P (Population) | Adult (age >18) patients with recurrent traumatic **anterior** shoulder instability |  |
| I (Intervention) | Latarjet or Bristow procedures, or their variants, including congruent arc modification | Stabilising operations which did not involve a coracoid process transfer with the conjoint tendon providing a sling effect (e.g. free coracoid graft without conjoint tendon) |
| C (Comparison) | Alternative free bone block procedures **involving bone grafts**, such as:   - iliac crest bone graft - free coracoid graft without conjoint tendon - J-bone graft - distal tibia allograft - distal clavicle autograft - spine of scapula autograft - any other allografts (glenoid, distal radius, etc.) | Procedures which did not involve the use of a bone block, e.g. arthroscopic stabilisation, and any only soft tissue stabilisations (e.g. Bankart +/- Remplissage) |
| O (Outcomes) | Primary outcome: **Rate of recurrent dislocation**  Secondary outcomes:  Patient-reported outcome measure indices/scores Radiological outcomes (e.g., secondary osteoarthritis) Pain and function (e.g., range of motion) Adverse outcomes (e.g., scapular dyskinesis rate, reoperation rate) | Minimum 12 months of post-operative follow-up for clinical studies. |
| S (Study design) | Studies **comparing** Latarjet with an alternative free bone block procedures. | Non-comparative studies. Case reports, abstracts, letters, editorials, reviews.  Comparisons of classic Latarjet vs congruent arc Latarjet, or open vs arthroscopic Latarjet without comparisons to another free bone block technique |
