# Supplementary material for: Clinical outcomes and complications in Latarjet versus free bone block procedures for anterior shoulder instability: a meta-analysis of comparative studies
Source: Eur J Orthop Surg Traumatol. 2025 Aug 31;35(1):371. doi: 10.1007/s00590-025-04485-0 (PMC12399734; doi:10.1007/s00590-025-04485-0)
Supplement: Supplementary file 3 — Supplementary file3 (DOCX 31 kb) [file 590_2025_4485_MOESM3_ESM.docx]

**Supplementary Table S3** Characteristics of articles included in the meta-analysis. *DTA*: distal tibia allograft. *ICBG*: Iliac crest bone graft

| **Study Author(s)** | **Publication year** | **Study type** | **Level of evidence** | **Surgery indication** | **Total number of patients operated** | **Cohorts** | **Number of patients followed-up (% of treated)** | **Mean age (years)** | **Male/female sex, n** | **Hand dominance (operated on dominant hand)** | **Mean number of previous surgeries per patient** | **Mean number of instability episodes preoperatively** | **Primary vs Revision procedures** | **Mean glenoid bone loss** | **Mean length of follow up (months)** |
| --- | --- | --- | --- | --- | --- | --- | --- | --- | --- | --- | --- | --- | --- | --- | --- |
| Carbone et al. | 2016 | Retrospective cohort study | Level III | Recurrent anterior shoulder dislocation with minimum 20% glenoid bone loss | 46 | Overall | 40 (87.0%) | 25 | - | 30/40 (75%) | Excluded | - | Primary N = 40 (100%) | - | 20 (SD: 8; range: 12-60 months) |
|  |  |  |  |  |  |  |  |  |  |  |  |  | Revision N = 0 (0%) |  |  |
|  |  |  |  |  |  | Open Latarjet | 20 (87.0%) | 24 (SD: 8; range: 18-44) | - | 16/20 (80%) | Excluded | - | Primary N = 20 (100%) | - | - |
|  |  |  |  |  |  |  |  |  |  |  |  |  | Revision N = 0 (0%) |  |  |
|  |  |  |  |  |  | Open J-bone graft (modified ICBG) | 20 (87.0%) | 26 (SD: 17; range: 8-60 ) | - | 14/20 (70%) | Excluded | - | Primary N = 20 (100%) | - | - |
|  |  |  |  |  |  |  |  |  |  |  |  |  | Revision N = 0 (0%) |  |  |
|  |  |  |  |  |  | (P-value) | - | P = .62 | - | - | - | - | - | - | - |
| Frank et al. | 2018 | Prospective cohort study | Level III | Recurrent anterior shoulder instability (dislocation or subluxation) with minimum 15% glenoid bone loss | 100 | Overall | 100 (100.0%) | 25.61 (SD: 6.09) | 96M/4F | - | 0.93 | - | Primary N = 36 (36%) | 25.51% (SD: 8.86) | 45 (SD: 20; range: 24-111 months) |
|  |  |  |  |  |  |  |  |  |  |  |  |  | Revision N = 64 (64%) |  |  |
|  |  |  |  |  |  | Open Latarjet | 50 (100.0%) | 25.40 (SD: 6.29) | 48M/2F | - | 0.88 (SD: 0.80) | - | Primary N = 18 (36%) | 22.40% (SD: 10.31) | - |
|  |  |  |  |  |  |  |  |  |  |  |  |  | Revision N = 32 (64%) |  |  |
|  |  |  |  |  |  | Open DTA | 50 (100.0%) | 25.82 (SD: 5.90) | 48M/2F | - | 0.98 (SD: 0.89) | - | Primary N = 18 (36%) | 28.62% (SD: 7.41) | - |
|  |  |  |  |  |  |  |  |  |  |  |  |  | Revision N = 32 (64%) |  |  |
|  |  |  |  |  |  | (P-value) | - | P = .731 | P = .691 | - | P = .556 | - | - | P = .001 | - |
| Wong et al. | 2018 | Retrospective cohort study | Level III | Anterior shoulder instability with bone loss with or without previous failed surgery | 48 | Overall | 48 (100.0%) | 29.7 (range: 18-70 ) | 32M/16F | - | - | - | Primary N = 37 (77%) | 19.73% | 26.29 |
|  |  |  |  |  |  |  |  |  |  |  |  |  | Revision N = 11 (23%) |  |  |
|  |  |  |  |  |  | Arthroscopic Latarjet (“coracoid transfer”) | 12 (100.0%) | - | - | - | - | - | Primary N = 11 (91.7%) | 18.69% (SD: 11.36%) | 26.79 (SD: 3.9 months) |
|  |  |  |  |  |  |  |  |  |  |  |  |  | Revision N = 1 (8.3%) |  |  |
|  |  |  |  |  |  | Arthroscopic DTA | 36 (100.0%) | - | - | - | - | - | Primary N = 26 (72.2%) | 20.08% (SD: 8.59%) | 26.12 (SD: 4.3 months) |
|  |  |  |  |  |  |  |  |  |  |  |  |  | Revision N = 10 (27.8%) |  |  |
|  |  |  |  |  |  | (P-value) | - | - | - | - | - | - | - | P = .65 | - |
| Moroder et al. | 2019 | Randomized controlled trial | Level II | Anterior shoulder instability with recurrent dislocations (over 1) with minimum 15% glenoid bone loss | 60 | Overall | 54 (90.0%) | 30 | 56M/4F | - | 0.69 |  | Primary N = 30 (50%) | - | 24 |
|  |  |  |  |  |  |  |  |  |  |  |  |  | Primary N = 30 (50%) |  |  |
|  |  |  |  |  |  | Open Latarjet | 25 (83.3%) | 31 (SD: 8; range: 18-47) | 28M/2F | - | 0.68 | 16 (SD: 18; range: 2-80) | Primary N = 15 (50%) | - | - |
|  |  |  |  |  |  |  |  |  |  |  |  |  | Revision N = 15 (50%) |  |  |
|  |  |  |  |  |  | Open J-bone graft (ICBG) | 29 (96.7%) | 29 (SD: 9; range: 18-57) | 28M/2F | - | 0.69 | 19 (SD: 19; range: 3-80) | Primary N = 17 (56.7%) | - | - |
|  |  |  |  |  |  |  |  |  |  |  |  |  | Revision N = 13 (43.3%) |  |  |
|  |  |  |  |  |  | (P-value) | - | P = .446 | P > .999 | - | P = .954 | P = .263 | P = .353 | - | - |
| Mahmoud et al. | 2022 | Prospective cohort study | Level III | Recurrent anterior shoulder instability with glenoid defect >20% | 50 | Overall | 50 (100.0%) | 26.7 | 45M/5F | 40/50 (80%) | - | - | Primary N = 0 (0%) | 26.5 | 50.85 |
|  |  |  |  |  |  |  |  |  |  |  |  |  | Revision N = 50 (100%) |  |  |
|  |  |  |  |  |  | “Mini-open” Latarjet | 25 (100.0%) | 25.7 (SD: 5.9) | 23M/2F | 19/25 (76%) | - | - | Primary N = 0 (0%) | 26.2 (SD: 2.7) | 50.1 (SD: 5.9; range: 38-59) |
|  |  |  |  |  |  |  |  |  |  |  |  |  | Revision N = 25 (100%) |  |  |
|  |  |  |  |  |  | Arthroscopic tricortical ICBG | 25 (100.0%) | 27.7 (SD: 4.7) | 22M/3F | 21/25 (84%) | - | - | Primary N = 0 (0%) | 26.8 (SD: 3.9) | 51.6 (SD: 6.8; range: 36-61) |
|  |  |  |  |  |  |  |  |  |  |  |  |  | Revision N = 25 (100%) |  |  |
|  |  |  |  |  |  | (P-values) | - | - | P = 1 | - | - | - | - | P = .51 | - |
| Razaeian et al. | 2022 | Retrospective cohort study | Level III | Recurrent anterior-inferior shoulder instability with glenoid defect >13.5% | 64 | Overall | 43 (67.2%) | 29.9 | 39M/4F | 25/43 (58%) | - | - | Primary N = 28 (65.1%) | 22.1 | 34.9 (range: 22-66 months) |
|  |  |  |  |  |  |  |  |  |  |  |  |  | Revision N = 15 (34.9%) |  |  |
|  |  |  |  |  |  | Open Latarjet | 21 (75.0%) | 27.19 (SD: 7.17) | 19M/2F | 16/21 (76%) | - | 10.8 (range: 2-50) | Primary N = 13 (61.9%) | 22.9% (range: 14-27%) | 45.5 (SD: 13) |
|  |  |  |  |  |  |  |  |  |  |  |  |  | Revision N = 8 (38.1%) |  |  |
|  |  |  |  |  |  | All-arthroscopic autologous tricortical ICBG | 22 (61.1%) | 32.55 (SD: 8.83) | 20M/2F | 9/22 (41%) | - | 24.2 (2-100) | Primary N = 15 (68.2%) | 21.3% (range: 14-27) | 24.8 (SD: 2.4) |
|  |  |  |  |  |  |  |  |  |  |  |  |  | Revision N = 7 (31.8%) |  |  |
|  |  |  |  |  |  | (P-value) | - | P = .03 | P = 1 | - | - | P = .14 | P = .45 | P = .17 | P < 0.001 |

| **Study Author(s)** | **Publication year** | **Study type** | **Level of evidence** | **Surgery indication** | **Total number of patients operated** | **Cohorts** | **Number of patients followed-up (% of treated)** | **Mean age (years)** | **Male/female sex, n** | **Hand dominance (operated on dominant hand)** | **Mean number of previous surgeries per patient** | **Mean number of instability episodes preoperatively** | **Primary vs Revision procedures** | **Mean glenoid bone loss** | **Mean length of follow up (months)** |
| --- | --- | --- | --- | --- | --- | --- | --- | --- | --- | --- | --- | --- | --- | --- | --- |
| Bockmann et al. | 2023 | Prospective cohort study | Level III | Recurrent anterior shoulder instability with glenoid defect >10% | 177 | Overall | 133 (75.1%) | 29 (SD: 8; 95% CI: 28-30; range: 18-57) | 144M/30F | - | 0.6 (range: 0-4) | 15 (SD: 20; 95% CI: 12-22; range: 1-100) | Primary N = 105 (60%) | 19% (SD: 5; 95% CI: 18-20; range: 10-40) | 44 |
|  |  |  |  |  |  |  |  |  |  |  |  |  | Revision N = 70 (40%) |  |  |
|  |  |  |  |  |  | Arthroscopic Latarjet | 78 (71%) | 28 (SD: 8; 95% CI: 27-30; range: 18-52) | 86M/22F | - | 0.5 (range: 0-2) | 14 (SD: 20; 95% CI: 10-18; range: 1-100) | Primary N = 72 (65.5%) | 18% (SD: 5; 95% CI: 17-19; range: 10-25) | 46 (SD: 15) |
|  |  |  |  |  |  |  |  |  |  |  |  |  | Revision N = 38 (34.5%) |  |  |
|  |  |  |  |  |  | Arthroscopic ICBG | 55 (82%) | 32 (SD: 10; 95% CI: 29-36; range: 20-57) | 58M/8F | - | 0.7 (range: 0-4) | 17 (SD: 21; 95% CI: 12-22; range: 1-100) | Primary N = 33 (50.8%) | 21% (SD: 6; 95% CI: 19-22; range: 11-40) | 41 (SD: 14) |
|  |  |  |  |  |  |  |  |  |  |  |  |  | Revision N = 32 (49.2%) |  |  |
|  |  |  |  |  |  | (P-value) | - | P = .031 | NS | - | NS | NS | NS | - | NS |
| Hussine et al. | 2023 | Randomised controlled trial | Level II | Recurrent anterior shoulder instability with >15% glenoid bone loss | 40 | Overall | 40 | 25.9 (SD, 5.6; range, 18-38) | 36M/4F | - |  | 11.95 (SD, 5.38; range, 5-21) | - | - | 16 months (SD, 3.4; range, 8-24) |
|  |  |  |  |  |  |  |  |  |  |  |  |  |  |  |  |
|  |  |  |  |  |  | Open Latarjet | 20 | - | - | - |  | - | - | - | - |
|  |  |  |  |  |  |  |  |  |  |  |  |  |  |  |  |
|  |  |  |  |  |  | Open ICBG | 20 | - | - | - |  | - | - | - | - |
|  |  |  |  |  |  |  |  |  |  |  |  |  |  |  |  |
|  |  |  |  |  |  | (P-value) | - | - | - | - |  | - | - | - | - |
| Delgado et al. | 2024 | Prospective cohort study | Level III | Anterior glenohumeral instability and glenoid bone loss >5% | 40 | Overall | 40 (100%) | 29.5 (SD, 7.9) | 36M/4F | 20/40 (50%) | - | - | Primary N = 40 (100%) | 15.2% (SD, 3.9) | Final follow-up 33.3 (SD, 10.1) |
|  |  |  |  |  |  |  |  |  |  |  |  |  | Revision N = 0 (0%) |  |  |
|  |  |  |  |  |  | Latarjet Overall | 20 (100%) | - | 16M/4F | 12/20 (60%) | - | - | Primary N = 20 (100%) | 17.35% | - |
|  |  |  |  |  |  |  |  |  |  |  |  |  | Revision N = 0 (0%) |  |  |
|  |  |  |  |  |  | Open Latarjet | 10 (100%) | Median 37 (IQR, 22-52) | 8M/2F | 5/10 (50%) | - | - | Primary N = 10 (100%) | 18.6% (SD, 3.05) | - |
|  |  |  |  |  |  |  |  |  |  |  |  |  | Revision N = 0 (0%) |  |  |
|  |  |  |  |  |  |  |  |  |  |  |  |  |  |  |  |
|  |  |  |  |  |  | Arthroscopic Latarjet | 10 (100%) | Median 22 (IQR, 23-58) | 8M/2F | 7/10 (70%) | - | - | Primary N = 10 (100%) | 16.1% (SD, 4.48) | - |
|  |  |  |  |  |  |  |  |  |  |  |  |  | Revision N = 0 (0%) |  |  |
|  |  |  |  |  |  |  |  |  |  |  |  |  |  |  |  |
|  |  |  |  |  |  | Arthroscopic ICBG Overall | 20 (100%) | - | 20M/0F | 8/20 (40%) | - | - | Primary N = 20 (100%) | 13.10% | - |
|  |  |  |  |  |  |  |  |  |  |  |  |  | Revision N = 0 (0%) |  |  |
|  |  |  |  |  |  |  |  |  |  |  |  |  |  |  |  |
|  |  |  |  |  |  | Arthroscopic ICBG - Allograft | 10 (100%) | Median 26 (IQR, 18-35) | 10M/0F | 5/10 (50%) | - | - | Primary N = 10 (100%) | 12.2% (SD, 1.99) | - |
|  |  |  |  |  |  |  |  |  |  |  |  |  | Revision N = 0 (0%) |  |  |
|  |  |  |  |  |  |  |  |  |  |  |  |  |  |  |  |
|  |  |  |  |  |  | Arthroscopic ICBG - Autograft | 10 (100%) | Median 28 (IQR, 22-55) | 10M/0F | 3/10 (30%) | - | - | Primary N = 10 (100%) | 14% (SD, 2.62) | - |
|  |  |  |  |  |  |  |  |  |  |  |  |  | Revision N = 0 (0%) |  |  |
|  |  |  |  |  |  | (P-value) | - | P = 0.608 | P = 0.939 | P = 0.362 | - | - | - | P = 0.015 | - |
| Elwan et al. | 2024 | Randomised controlled trial | Level II | Recurrent shoulder dislocation with glenoid bone loss of more than 20% loss, off‑track Hill‑Sachs lesions, or combined bipolar lesions | 40 | Overall | 40 (100%) | 25.8 | 35M/5F | 32/40 (80%) | - | - | Primary N = 34 (85%) | <20%: 7 20-33%: 25 30-45%: 8 | At least 12 months follow-up |
|  |  |  |  |  |  |  |  |  |  |  |  |  | Revision N = 6 (15%) |  |  |
|  |  |  |  |  |  | Open Latarjet | 20 (100%) | 26.5 (SD, 5.68) | 16M/4F | 14/20 (70%) | - | 14/20 had >10 episodes of dislocation | Primary N = 18 (90%) | <20%: 4 20-33%: 13 30-45%: 3 | - |
|  |  |  |  |  |  |  |  |  |  |  |  |  | Revision N = 2 (10%) |  |  |
|  |  |  |  |  |  | Open ICBG | 20 (100%) | 25.05 (SD, 4.57) | 19M/1F | 18/20 (90%) | - | 16/20 had >10 episodes of dislocation | Primary N = 16 (80%) | <20%: 3 20-33%: 12 30-45%: 5 | - |
|  |  |  |  |  |  |  |  |  |  |  |  |  | Revision N = 4 (20%) |  |  |
|  |  |  |  |  |  | (P-value) | - | P = 0.495 | P = 0.342 | P = 0.235 | - | 0.456 | 0.661 | 0.709 | - |
| Schulz et al. | 2025 | Randomised controlled trial | Level II | Anterior shoulder instability with recurrent dislocations with minimum 15% glenoid bone loss | 60 | Overall | 39 (65%) | See data in Moroder et al. | | | | | | | 60 months |
|  |  |  |  |  |  |  |  |  |  |  |  |  |  |  |  |
|  |  |  |  |  |  | Open Latarjet | 20 (67%) |  |  |  |  |  |  |  | - |
|  |  |  |  |  |  |  |  |  |  |  |  |  |  |  |  |
|  |  |  |  |  |  | Open J-bone graft (ICBG) | 19 (63%) |  |  |  |  |  |  |  | - |
|  |  |  |  |  |  |  |  |  |  |  |  |  |  |  |  |
|  |  |  |  |  |  | (P-value) | - |  |  |  |  |  |  |  | - |
