# Supplementary material for: Clinical outcomes and complications in Latarjet versus free bone block procedures for anterior shoulder instability: a meta-analysis of comparative studies
Source: Eur J Orthop Surg Traumatol. 2025 Aug 31;35(1):371. doi: 10.1007/s00590-025-04485-0 (PMC12399734; doi:10.1007/s00590-025-04485-0)
Supplement: Supplementary file 4 — Supplementary file4 (DOCX 26 kb) [file 590_2025_4485_MOESM4_ESM.docx]

**Supplementary Table S4** Recurrence rate by study. P-values as reported. *DTA*: distal tibia allograft. *ICBG*: Iliac crest bone graft.

| **Study Author(s)** | **Cohort** | **Recurrence rate** |  |
| --- | --- | --- | --- |
| Carbone et al. | Open Latarjet | Not reported |  |
|  |  |  |  |
|  |  |  |  |
|  | Open J-bone graft (modified ICBG) | Not reported |  |
|  |  |  |  |
|  |  |  |  |
|  | (P-value) | - |  |
| Frank et al. | Open Latarjet | 0/50 (0%) |  |
|  |  |  |  |
|  |  |  |  |
|  | Open DTA | 1/50 (2%) |  |
|  |  |  |  |
|  |  |  |  |
|  | (P-value) | - |  |
| Wong et al. | Arthroscopic Latarjet (“coracoid transfer”) | 2/12 (16%) |  |
|  |  |  |  |
|  |  |  |  |
|  | Arthroscopic DTA | 2/36 (6%) |  |
|  |  |  |  |
|  |  |  |  |
|  | (P-value) | P = 0.25 |  |
| Mahmoud et al. | “Mini-open” Latarjet | 1/25 (4%) |  |
|  |  |  |  |
|  |  |  |  |
|  | Arthroscopic tricortical ICBG | 0/25 (0%) |  |
|  |  |  |  |
|  |  |  |  |
|  | (P-value) | - |  |
| Razaeian et al. | Open Latarjet | 1 (4.8%) |  |
|  |  |  |  |
|  |  |  |  |
|  | Arthroscopic autologous tricortical ICBG | 1 (4.5%) |  |
|  |  |  |  |
|  |  |  |  |
|  | (P-value) | - |  |
| Bockmann et al. | Arthroscopic Latarjet | 4/78 (5.1%) |  |
|  |  |  |  |
|  |  |  |  |
|  | Arthroscopic ICBG | 3/55 (5.5%) |  |
|  |  |  |  |
|  |  |  |  |
|  | (P-value) | - |  |
| Hussine et al. | Open Latarjet | 0 instability events |  |
|  |  |  |  |
|  |  |  |  |
|  | Open ICBG | 0 instability events |  |
|  |  |  |  |
|  |  |  |  |
|  | (P-value) | - |  |
| Delgado et al. | Latarjet - Overall | 0 (0%) |  |
|  |  |  |  |
|  |  |  |  |
|  | Open Latarjet | 0 (0%) |  |
|  |  |  |  |
|  |  |  |  |
|  | Arthroscopic Latarjet | 0 (0%) |  |
|  |  |  |  |
|  |  |  |  |
|  | ICBG - Overall | 1 (5%) |  |
|  |  |  |  |
|  |  |  |  |
|  | ICBG - Allograft | 1 (10%) |  |
|  |  |  |  |
|  |  |  |  |
|  | ICBG - Autograft | 0 (0%) |  |
|  |  |  |  |
|  |  |  |  |
|  | (P-value) | - |  |
| Elwan et al. | Open Latarjet | 1 dislocation due to tramadol seizure |  |
|  |  |  |  |
|  |  |  |  |
|  | Open ICBG | 0 |  |
|  |  |  |  |
|  |  |  |  |
|  | (P-value) | - |  |
| Schulz et al. | Open Latarjet | 1 (traumatic subluxation) |  |
|  |  |  |  |
|  |  |  |  |
|  | Open J-bone graft (ICBG) | 3 (2 traumatic isolated subluxations, 1 traumatic dislocation with glenoid rim fracture) |  |
|  |  |  |  |
|  |  |  |  |
|  | (P-value) | P=0.342 |  |
