# Supplementary material for: Clinical outcomes and complications in Latarjet versus free bone block procedures for anterior shoulder instability: a meta-analysis of comparative studies
Source: Eur J Orthop Surg Traumatol. 2025 Aug 31;35(1):371. doi: 10.1007/s00590-025-04485-0 (PMC12399734; doi:10.1007/s00590-025-04485-0)
Supplement: Supplementary file 5 — Supplementary file5 (DOCX 23 kb) [file 590_2025_4485_MOESM5_ESM.docx]

**Supplementary Table S5** Patient-reported outcome measures by study. *DTA*: distal tibia allograft. *ICBG*: Iliac crest bone graft.

| **Study Author(s)** | **Cohort** | **Pre-op/Post-op** | **Mean WOSI score** | **Mean Rowe score** | **Mean SSV** | **Mean SANE score** | **Mean SST score** | **Mean VAS value** | **Mean ASES score** | **Mean UCLA score** | **Mean Constant score** | **Mean SSI value** |
| --- | --- | --- | --- | --- | --- | --- | --- | --- | --- | --- | --- | --- |
| Carbone et al. | Open Latarjet | Pre-op | - | - | - | - | - | - | - | - | - | - |
|  |  | Post-op | 259 (SD, 164; range, 30- 528) | 87 (SD, 8; range, 70-100) | 88 (SD, 10; range, 70-100) | - | - | - | - | - | - | - |
|  |  | Pre-op/Post-op P-value | - | - | - | - | - | - | - | - | - | - |
|  | Open J-bone graft (modified ICBGT) | Pre-op | - | - | - | - | - | - | - | - | - | - |
|  |  | Post-op | 252 (SD 182; range: 8-576; P = 0.43) | 91 (SD, 8; range, 73-100; P = 0.09) | 90 (SD, 9; range, 70-100; P = 0.21) | - | - | - | - | - | - | - |
|  |  | Pre-op/Post-op P-value | - | - | - | - | - | - | - | - | - | - |
|  | (post-op P-value between cohorts) | | P = 0.43 | P = 0.09 | P = 0.21 | - | - | - | - | - | - | - |
| Frank et al. | Open Latarjet | Pre-op | 39.8 (SD, 22.0) | - | - | 29.1 (SD,18.5) | 58.0 (SD, 31.5) | 2.8 (SD, 2.7) | 65.5 (SD, 20.9) | - | - | - |
|  |  | Post-op | 74.30 (SD, 21.84) | - | - | 80.68 (SD, 7.21) | 94.84 (SD, 7.12) | 0.67 (SD, 0.97) | 91.06 (SD, 8.78) | - | - | - |
|  |  | Pre-op/Post-op P-value | - | - | - | - | - | - | - | - | - | - |
|  | Open DTA | Pre-op | 32.9 (SD, 13.7) | - | - | 42.5 (SD, 19.8) | 58.3 (SD, 27.7) | 3.1 (SD, 2.9) | 54.8 (SD, 20.8) | - | - | - |
|  |  | Post-op | 89.69 (SD, 5.50) | - | - | 90.08 (SD, 13.39) | 86.57 (SD, 18.99) | 1.83 (SD, 2.31) | 89.74 (SD, 12.66) | - | - | - |
|  |  | Pre-op/Post-op P-value | - | - | - | - | - | - | - | - | - | - |
|  | (post-op P-value between cohorts) | | P = 0.537 | - | - | P = 0.058 | P = 0.011 | P = 0.092 | P = 0.127 | - | - | - |
| Wong et al. | Arthroscopic Latarjet (“coracoid transfer”) | Pre-op | - | - | - | - | - | - | - | - | - | - |
|  |  | Post-op | - | - | - | - | - | - | - | - | - | - |
|  |  | Pre-op/Post-op P-value | - | - | - | - | - | - | - | - | - | - |
|  | Arthroscopic DTA | Pre-op | - | - | - | - | - | - | - | - | - | - |
|  |  | Post-op | - | - | - | - | - | - | - | - | - | - |
|  |  | Pre-op/Post-op P-value | - | - | - | - | - | - | - | - | - | - |
|  | (post-op P-value between cohorts) | | - | - | - | - | - | - | - | - | - | - |
| Mahmoud et al. | “Mini-open” Latarjet | Pre-op | - | - | - | - | - | - | - | 14.2 (SD, 3.3) | 51 (SD, 5.5) | - |
|  |  | Post-op | - | - | - | - | - | - | - | 31.1 (SD, 2.1) | 90.2 (SD, 4.6) | - |
|  |  | Pre-op/Post-op P-value | - | - | - | - | - | - | - | - | - | - |
|  | Arthroscopic tricortical ICBG | Pre-op | - | - | - | - | - | - | - | 15.4 (SD, 3.1) | 53.6 (SD, 6.04) | - |
|  |  | Post-op | - | - | - | - | - | - | - | 30.2 (SD, 2.2) | 89 (SD, 5.01) | - |
|  |  | Pre-op/Post-op P-value | - | - | - | - | - | - | - | - | - | - |
|  | (post-op P-value between cohorts) | | - | - | - | - | - | - | - | P = 0.17 | P = 0.38 | - |
| Razaeian et al. | Open Latarjet | Pre-op | - | - | - | - | - | - | - | - | - | - |
|  |  | Post-op | 45 (SD, 31.3) | 84.8 (SD, 12.7) | 83.8 (SD, 11.17) | - | - | 1.6 (SD, 2) | - | - | - | 7.6 (SD 11.3) |
|  |  | Pre-op/Post-op P-value | - | - | - | - | - | - | - | - | - | - |
|  | Arthroscopic autologous tricortical ICBG | Pre-op | - | - | - | - | - | - | - | - | - | - |
|  |  | Post-op | 32.1 (SD, 33.1) | 93 (SD, 10.7) | 89.1 (SD, 10.8) | - | - | 1.0 (SD, 1.3) | - | - | - | 6.8 (SD, 10.4) |
|  |  | Pre-op/Post-op P-value | - | - | - | - | - | - | - | - | - | - |
|  | (post-op P-value between cohorts) | | P = 0.07 | P = 0.008 | P = 0.12 | - | - | P = 0.37 | - | - | - | P = 0.91 |
| Bockmann et al. | Arthroscopic Latarjet | Pre-op | 47 (SD, 18) | 49 (SD, 22) | 55 (SD, 22) | - | - | - | - | - | - | - |
|  |  | Post-op | 78 (SD, 20) | 85 (SD, 21) | 86 (SD, 13) | - | - | - | - | - | - | - |
|  |  | Pre-op/Post-op P-value | - | - | - | - | - | - | - | - | - | - |
|  | Arthroscopic ICBG | Pre-op | 46 (SD, 17) | 34 (SD, 19) | 53 (SD, 21) | - | - | - | - | - | - | - |
|  |  | Post-op | 75 (SD, 21) | 84 (SD, 19) | 83 (SD, 18) | - | - | - | - | - | - | - |
|  |  | Pre-op/Post-op P-value | - | - | - | - | - | - | - | - | - | - |
|  | (post-op P-value between cohorts) | | - | - | - | - | - | - | - | - | - | - |
| Hussine et al. | Open Latarjet | Pre-op | - | 18 (SD, 11; range, 5-40) | - | - | - | - | 40 (SD, 11; range, 20-58) | - | - | - |
|  |  | Post-op | - | 87 (SD, 10; range, 75-100) | - | - | - | - | 91 (SD, 8; range, 78-98) | - | - | - |
|  |  | Pre-op/Post-op P-value | - | - | - | - | - | - | - | - | - | - |
|  | Open ICBG | Pre-op | - | 23 (SD, 10; range, 5-45) | - | - | - | - | 35 (SD, 11; range, 20-58) | - | - | - |
|  |  | Post-op | - | 92 (SD, 10; range, 70-100) | - | - | - | - | 91 (SD, 7; range, 78-100) | - | - | - |
|  |  | Pre-op/Post-op P-value | - | - | - | - | - | - | - | - | - | - |
|  | (post-op P-value between cohorts) | | - | P = 0.14 | - | - | - | - | P = 0.54 | - | - | - |

Continued.

| **Study Author(s)** | **Cohort** | **Pre-op/Post-op** | **Mean WOSI score** | **Mean Rowe score** | **Mean SSV** | **Mean SANE score** | **Mean SST score** | **Mean VAS value** | **Mean ASES score** | **Mean UCLA score** | **Mean Constant score** | **Mean SSI value** |
| --- | --- | --- | --- | --- | --- | --- | --- | --- | --- | --- | --- | --- |
| Delgado et al. | Latarjet - Overall | Pre-op | - | - | - | - | - | - | - | - | - | - |
|  |  | Post-op | - | - | - | - | - | - | - | - | - | - |
|  |  | Pre-op/Post-op P-value | - | - | - | - | - | - | - | - | - | - |
|  | Open Latarjet | Pre-op | - | - | - | - | - | - | - | - | - | - |
|  |  | Post-op | - | - | - | - | - | - | - | - | - | - |
|  |  | Pre-op/Post-op P-value | - | - | - | - | - | - | - | - | - | - |
|  | Arthroscopic Latarjet | Pre-op | - | - | - | - | - | - | - | - | - | - |
|  |  | Post-op | - | - | - | - | - | - | - | - | - | - |
|  |  | Pre-op/Post-op P-value | - | - | - | - | - | - | - | - | - | - |
|  | ICBG - Overall | Pre-op | - | - | - | - | - | - | - | - | - | - |
|  |  | Post-op | - | - | - | - | - | - | - | - | - | - |
|  |  | Pre-op/Post-op P-value | - | - | - | - | - | - | - | - | - | - |
|  | ICBG - Allograft | Pre-op | - | - | - | - | - | - | - | - | - | - |
|  |  | Post-op | - | - | - | - | - | - | - | - | - | - |
|  |  | Pre-op/Post-op P-value | - | - | - | - | - | - | - | - | - | - |
|  | ICBG - Autograft | Pre-op | - | - | - | - | - | - | - | - | - | - |
|  |  | Post-op | - | - | - | - | - | - | - | - | - | - |
|  |  | Pre-op/Post-op P-value | - | - | - | - | - | - | - | - | - | - |
|  | (post-op P-value between cohorts) | | - | - | - | - | - | - | - | - | - | - |
| Elwan et al. | Open Latarjet | Pre-op | - | 37.75 (SD, 17.51) | - | - | - | - | - | - | - | - |
|  |  | Post-op | - | 86.25 (SD, 12.55) | - | - | - | - | - | - | - | - |
|  |  | Pre-op/Post-op P-value | - | - | - | - | - | - | - | - | - | - |
|  | Open ICBG | Pre-op | - | 36.75 (SD, 17.86) | - | - | - | - | - | - | - | - |
|  |  | Post-op | - | 87.75 (SD, 13.5) | - | - | - | - | - | - | - | - |
|  |  | Pre-op/Post-op P-value | - | - | - | - | - | - | - | - | - | - |
|  | (post-op P-value between cohorts) | | - | P = 0.699 | - | - | - | - | - | - | - | - |
| Schulz et al. | Open Latarjet | Pre-op | - | - | - | - | - | - | - | - | - | - |
|  |  | Post-op | 201 (SD, 239) | 94 (SD, 9) | 89 (SD, 14) | - | - | 0.3 (SD, 0.5) | - | - | - | - |
|  |  | Pre-op/Post-op P-value | - | - | - | - | - | - | - | - | - | - |
|  | Open J-bone graft (ICBG) | Pre-op | - | - | - | - | - | - | - | - | - | - |
|  |  | Post-op | 221 (SD, 186) | 93 (SD, 12) | 88 (SD, 13) | - | - | 0.2 (SD, 0.4) | - | - | - | - |
|  |  | Pre-op/Post-op P-value | - | - | - | - | - | - | - | - | - | - |
|  | (post-op P-value between cohorts) | | P=0.529 | P=0.596 | P=0.368 | - | - | P = 0.238 | - | - | - | - |
