# Supplementary material for: Clinical outcomes and complications in Latarjet versus free bone block procedures for anterior shoulder instability: a meta-analysis of comparative studies
Source: Eur J Orthop Surg Traumatol. 2025 Aug 31;35(1):371. doi: 10.1007/s00590-025-04485-0 (PMC12399734; doi:10.1007/s00590-025-04485-0)
Supplement: Supplementary file 6 — Supplementary file6 (DOCX 18 kb) [file 590_2025_4485_MOESM6_ESM.docx]

**Supplementary Table S6** Range of motion by study. *DTA*: distal tibia allograft. *ICBG*: Iliac crest bone graft

| **Study Author(s)** | **Cohort** | **Abduction** | **Forward flexion** | **External rotation** | **Internal rotation** | **Extension** |  |
| --- | --- | --- | --- | --- | --- | --- | --- |
| Carbone et al. | Open Latarjet | - | - | - | - | - |  |
|  |  |  |  |  |  |  |  |
|  |  |  |  |  |  |  |  |
|  | Open J-bone graft (modified ICBG) | - | - | - | - | - |  |
|  |  |  |  |  |  |  |  |
|  |  |  |  |  |  |  |  |
|  | (P-value) | - | - | - | - | - |  |
| Frank et al. | Open Latarjet | 153.50 (SD, 22.55) | 166.61 (SD, 15.80) | 74.86 (SD, 11.06) | 54.53 (SD, 14.97) | 58.22 (SD, 14.50) |  |
|  |  |  |  |  |  |  |  |
|  |  |  |  |  |  |  |  |
|  | Open DTA | 137.57 (SD, 44.34) | 156.84 (SD, 27.61) | 81.53 (SD, 14.64) | 53.87 (SD, 17.82) | 50.25 (SD, 23.74) |  |
|  |  |  |  |  |  |  |  |
|  |  |  |  |  |  |  |  |
|  | (P-value) | P = 0.242 | P = 0.185 | P = 0.180 | P = 0.912 | P = 0.216 |  |
| Wong et al. | Arthroscopic Latarjet (“coracoid transfer”) | - | - | - | - | - |  |
|  |  |  |  |  |  |  |  |
|  |  |  |  |  |  |  |  |
|  | Arthroscopic DTA | - | - | - | - | - |  |
|  |  |  |  |  |  |  |  |
|  |  |  |  |  |  |  |  |
|  | (P-value) | - | - | - | - | - |  |
| Mahmoud et al. | "Mini-open" Latarjet | 166.4 (SD, 4.5) | 166.4 (SD, 5.1) | 81.3 (SD, 3.3) | 70.3 (SD, 5.2) | - |  |
|  |  |  |  |  |  |  |  |
|  |  |  |  |  |  |  |  |
|  | Arthroscopic tricortical ICBG | 164.3 (SD, 3.9) | 164.9 (SD, 6.1) | 79.6 (SD, 4.6) | 73.1 (SD, 6.2) | - |  |
|  |  |  |  |  |  |  |  |
|  |  |  |  |  |  |  |  |
|  | (P-value) | P = 0.084 | P = 0.18 | P = 0.14 | P = 0.09 | - |  |
| Razaeian et al. | Open Latarjet | - | - | - | - | - |  |
|  |  |  |  |  |  |  |  |
|  |  |  |  |  |  |  |  |
|  | All-arthroscopic autologous tricortical ICBG | - | - | - | - | - |  |
|  |  |  |  |  |  |  |  |
|  |  |  |  |  |  |  |  |
|  | (P-value) | - | - | - | - | - |  |
| Bockmann et al. | Arthroscopic Latarjet | - | - | 69 (SD, 15) | 64 (SD, 18) | - |  |
|  |  |  |  |  |  |  |  |
|  |  |  |  |  |  |  |  |
|  | Arthroscopic ICBG | - | - | 63 (SD, 17) | 66 (SD, 15) | - |  |
|  |  |  |  |  |  |  |  |
|  |  |  |  |  |  |  |  |
|  | (P-value) | - | - | P = 0.012 | NS | - |  |
| Hussine et al. | Open Latarjet | - | 169 (SD, 5) | At 0° abduction: 37 (SD, 5) At 90° abduction: 75 (SD, 5) | At 90° abduction: 86 (SD, 2) | - |  |
|  |  |  |  |  |  |  |  |
|  |  |  |  |  |  |  |  |
|  | Open ICBG | - | 172 (SD, 6) | At 0° abduction: 44 (SD, 7) At 90° abduction: 80 (SD, 7) | At 90° abduction: 81 (SD, 6) | - |  |
|  |  |  |  |  |  |  |  |
|  |  |  |  |  |  |  |  |
|  | (P-value) | - | P = 0.17 | At 0° abduction: P = 0.001 At 90° abduction: P = 0.02 | P = 0.09 | - |  |
| Delgado et al. | Latarjet - Overall | - | - | - | - | - |  |
|  |  |  |  |  |  |  |  |
|  |  |  |  |  |  |  |  |
|  | Open Latarjet | - | - | - | - | - |  |
|  |  |  |  |  |  |  |  |
|  |  |  |  |  |  |  |  |
|  | Arthroscopic Latarjet | - | - | - | - | - |  |
|  |  |  |  |  |  |  |  |
|  |  |  |  |  |  |  |  |
|  | ICBG - Overall | - | - | - | - | - |  |
|  |  |  |  |  |  |  |  |
|  |  |  |  |  |  |  |  |
|  | ICBG - Allograft | - | - | - | - | - |  |
|  |  |  |  |  |  |  |  |
|  |  |  |  |  |  |  |  |
|  | ICBG - Autograft | - | - | - | - | - |  |
|  |  |  |  |  |  |  |  |
|  |  |  |  |  |  |  |  |
|  | (P-value) | - | - | - | - | - |  |
| Elwan et al. | Open Latarjet | - | - | - | - | - |  |
|  |  |  |  |  |  |  |  |
|  |  |  |  |  |  |  |  |
|  | Open ICBG | - | - | - | - | - |  |
|  |  |  |  |  |  |  |  |
|  |  |  |  |  |  |  |  |
|  | (P-value) | - | - | - | - | - |  |
| Schulz et al. | Open Latarjet | 178 (SD, 6) | 178 (SD, 4) | 69 (SD, 15) | Up to the 9th thoracal vertebra (SD, 3 vertebra) | - |  |
|  |  |  |  |  |  |  |  |
|  |  |  |  |  |  |  |  |
|  | Open J-bone graft (ICBG) | 180 (SD, 0) | 177 (SD, 6) | 63 (SD, 11) | Up to the 6th thoracal vertebra (SD, 2 vertebra) | - |  |
|  |  |  |  |  |  |  |  |
|  |  |  |  |  |  |  |  |
|  | (P-value) | P=0.294 | P=0.936 | 0.117 | P=0.004 | - |  |
