# Supplementary material for: Clinical outcomes and complications in Latarjet versus free bone block procedures for anterior shoulder instability: a meta-analysis of comparative studies
Source: Eur J Orthop Surg Traumatol. 2025 Aug 31;35(1):371. doi: 10.1007/s00590-025-04485-0 (PMC12399734; doi:10.1007/s00590-025-04485-0)
Supplement: Supplementary file 7 — Supplementary file7 (DOCX 26 kb) [file 590_2025_4485_MOESM7_ESM.docx]

**Supplementary Table S7** Adverse outcomes by study. *DTA*: distal tibia allograft. *ICBG*: Iliac crest bone graft

| **Study Author(s)** | **Cohort** | **Reoperation rate** | **Scapular dyskinesis** |
| --- | --- | --- | --- |
| Carbone et al. | Open Latarjet | - | 5/20 (25%) |
|  | Open J-bone graft (modified ICBG) | - | 0/20 (0%) |
|  | (P-value) | - | P=0.047 |
| Frank et al. | Open Latarjet | 3/50 (6%) | - |
|  | Open DTA | 3/50 (6%) | - |
|  | (P-value) | - | - |
| Wong et al. | Arthroscopic Latarjet (“coracoid transfer”) | - | - |
|  | Arthroscopic DTA | - | - |
|  | (P-value) | - | - |
| Moroder et al. | Open Latarjet | 1/25 (4%) | 4/25 (16%) |
|  | Open J-bone graft (ICBG) | 0/29 (0%) | 1/29 (3%) |
|  | (P-value) | - | - |
| Mahmoud et al. | "Mini-open" Latarjet | 1/25 (4%) | - |
|  | Arthroscopic tricortical ICBG | 0/25 (0%) | - |
|  | (P-value) | - | - |
| Razaeian et al. | Open Latarjet | 1 (4.8%) | - |
|  | All-arthroscopic autologous tricortical ICBG | 0 (0%) | - |
|  | (P-value) | - | - |
| Bockmann et al. | Arthroscopic Latarjet | 8/78 (10.3%) | - |
|  | Arthroscopic ICBG | 4/55 (7.3%) | - |
|  | (P-value) | - | - |
| Hussine et al. | Open Latarjet | 1 (5%) | - |
|  | Open ICBG | 0 (0%) | - |
|  | (P-value) | - | - |
| Delgado et al. | Latarjet - Overall | - | 0 (0%) |
|  | Open Latarjet | - | 0 (0%) |
|  | Arthroscopic Latarjet | - | 0 (0%) |
|  | ICBG - Overall | - | 1 (5%) |
|  | ICBG - Allograft | - | 1 (10%) |
|  | ICBG - Autograft | - | 0 (0%) |
|  | (P-value) | - | - |
| Elwan et al. | Open Latarjet | 2 (10%) | - |
|  | Open ICBG | 0 (0%) | - |
|  | (P-value) | - | - |
| Schulz et al. | Open Latarjet | 1 (screw irritation) | - |
|  | Open J-bone graft (ICBGT) | 1 (traumatic glenoid rim fracture with a fully integrated graft) | - |
|  | (P-value) | - | - |
